# Muscarinic Modulation of SK2-Type K^+^ Channels Promotes Intrinsic Plasticity in L2/3 Pyramidal Neurons of the Mouse Primary Somatosensory Cortex

**DOI:** 10.1523/ENEURO.0453-19.2020

**Published:** 2020-03-04

**Authors:** Daniel F. Gill, Christian Hansel

**Affiliations:** Department of Neurobiology, University of Chicago, Chicago, IL 60637

**Keywords:** engram, ensemble, excitability, learning, neocortex, pyramidal cell

## Abstract

Muscarinic acetylcholine receptors (mAChRs) inhibit small-conductance calcium-activated K^+^ channels (SK channels) and enhance synaptic weight via this mechanism. SK channels are also involved in activity-dependent plasticity of membrane excitability (“intrinsic plasticity”). Here, we investigate whether mAChR activation can drive SK channel-dependent intrinsic plasticity in L2/3 cortical pyramidal neurons. Using whole-cell patch-clamp recordings from these neurons in slices prepared from mouse primary somatosensory cortex (S1), we find that brief bath application of the mAChR agonist oxotremorine-m (oxo-m) causes long-term enhancement of excitability in wild-type mice that is not observed in mice deficient of SK channels of the SK2 isoform. Similarly, repeated injection of depolarizing current pulses into the soma triggers intrinsic plasticity that is absent from SK2 null mice. Intrinsic plasticity lowers spike frequency adaptation and attenuation of spike firing upon prolonged activation, consistent with SK channel modulation. Depolarization-induced plasticity is prevented by bath application of the protein kinase A (PKA) inhibitor H89, and the casein kinase 2 (CK2) inhibitor TBB, respectively. These findings point toward a recruitment of two known signaling pathways in SK2 regulation: SK channel trafficking (PKA) and reduction of the calcium sensitivity (CK2). Using mice with an inactivation of CaMKII (T305D mice), we show that intrinsic plasticity does not require CaMKII. Finally, we demonstrate that repeated injection of depolarizing pulses in the presence of oxo-m causes intrinsic plasticity that surpasses the plasticity amplitude reached by either manipulation alone. Our findings show that muscarinic activation enhances membrane excitability in L2/3 pyramidal neurons via a downregulation of SK2 channels.

## Significance Statement

Small-conductance, calcium-activated K^+^ channels are solely activated by calcium transients, typically associated with spike burst firing, and mediate a slow outward current. Just as AMPA receptor subunits, SK channels show activity-dependent plasticity, and their functional downregulation enhances excitability and prevents curtailing of prolonged spike firing. Here, we show that this form of “intrinsic plasticity” is promoted by the activation of muscarinic acetylcholine receptors (mAChRs), and requires the activation of protein kinase A (PKA) and casein kinase 2 (CK2). The activation of mAChRs enhances the plasticity amplitude obtained by co-application of a somatic depolarization protocol. These findings show that cholinergic signaling drives long-term enhancement of spike firing in cortical pyramidal neurons, and identify modulation of SK channels as an underlying mechanism.

## Introduction

Cholinergic signaling affects synaptic plasticity through the activation of muscarinic acetylcholine receptors (mAChRs; Bear and Singer, 1986; Kirkwood et al., 1999; Origlia et al., 2006; Zhao and Tzounopoulos, 2011; Dennis et al., 2016). At synapses onto hippocampal CA1 pyramidal neurons, activation of M1-type mAChRs enhances spine calcium transients and facilitates the induction of long-term potentiation (LTP). These effects are due to an M1 receptor-driven inhibition of small-conductance calcium-activated SK-type K^+^ channels (Buchanan et al., 2010; Giessel and Sabatini, 2010). SK conductances are exclusively activated by calcium, and may curtail dendritic potentials (Cai et al., 2004). Blockade of SK channels by the selective inhibitor apamin enhances spine calcium transients and promotes LTP in the hippocampus (Ngo-Anh et al., 2005; Lin et al., 2008), while reducing the probability for LTP induction at parallel fiber to Purkinje cell synapses in the cerebellum (Belmeguenai et al., 2010). It seems that M1 receptor activation achieves effects similar to apamin application via its capability to downregulate SK channels. Apamin reduces a medium-fast component of afterhyperpolarization (mAHP) currents that has a time constant of approximately 200 ms (Stocker et al., 1999; Villalobos et al., 2004; see also Madison et al., 1987), suggesting a role of SK channels in moderately slow regularity processes. Nevertheless, SK conductances open rapidly within 1 ms (τ ∼10 ms; Xia et al., 1998; Sah and Faber, 2002; Bond et al., 2004), which allows them to influence the peak amplitude of spine calcium transients and dendritic potentials.

SK channels can also be downregulated in a lasting manner on synaptic or neuronal activation, causing an increase in membrane excitability and spike firing (Sourdet et al., 2003; Belmeguenai et al., 2010). The question arises whether or not mAChR activation can trigger such “intrinsic plasticity.” In layer 5 pyramidal neurons of rat cortex, SK channel plasticity is triggered by the activation of type 5 metabotropic glutamate receptors (mGluRs; Sourdet et al., 2003). Like Group I mGluRs (type 1 and 5), some mAChR types (M1, M3, and M5) couple to Gαq proteins. Thus, it is conceivable that mAChR activation indeed drives SK plasticity, and with it, intrinsic plasticity. The available evidence supports this possibility. While to the best of our knowledge mAChR-activated SK plasticity has not been reported yet, it has been shown that muscarinic signaling may upregulate excitability and spike firing over prolonged periods (Santini et al., 2012; Joshi et al., 2016), even lasting for 40 min and more (Moore et al., 2009; Park and Spruston, 2012). Both mAChRs (the M1, M3, and M4 subtypes; Rossner et al., 1993) and SK channels (SK1,2 and 3 isoforms; Gymnopoulos et al., 2014) are expressed in layer 2/3 of the rat primary somatosensory cortex (S1). Using whole-cell patch-clamp recordings from L2/3 pyramidal neurons in slices of mouse S1 cortex, we here show that in these neurons mAChR activation triggers intrinsic plasticity, and that this plasticity depends on the availability of SK2-type K^+^ channels. We demonstrate that SK2 plasticity depends on the activation of protein kinase A (PKA) and casein kinase 2 (CK2), and that combined muscarinic and depolarization-dependent activation enhances the overall amplitude of plasticity, without reaching synergistic effects above the sum of both manipulations alone.

## Materials and Methods

### Pyramidal cell recordings *ex vivo*

Thalamocortical slices (350 μm) including S1 cortex were prepared (orientation described in Agmon and Connors, 1991) from young adolescent mice of either sex [postnatal day (P)25–P40] after isoflurane anesthesia and decapitation. This procedure is in accordance with the guidelines of the Animal Care and Use Committee of the University of Chicago. The slices were cut on a vibratome (Leica VT1000S) using ceramic blades. The slices were cut in a sucrose slicing solution containing the following: 185 mM sucrose, 2.5 mM KCl, 25 mM glucose, 25 mM NaHCO_3_, 1.2 mM NaH_2_PO_4_, 0.5 mM CaCl_2_, and 0.5 mM MgCl_2_, bubbled with 95% O_2_ and 5% CO_2_. Following slicing, the slices were kept in artificial CSF (ACSF) containing the following: 124 mM NaCl, 5 mM KCl, 1.25 mM NaH_2_PO_4_, 2 mM MgSO_4_, 2 mM CaCl_2_, 26 mM NaHCO_3_, and 10 mM D-glucose, bubbled with 95% O_2_ and 5% CO_2_. The slices were allowed to recover for at least 1 h, and were then transferred to a submerged recording chamber superfused with ACSF at near-physiological temperature (31–34°C). Whole-cell patch-clamp recordings were performed under visual control using a 40× water-immersion objective mounted on a Zeiss Axioskop 2FS microscope. Patch pipettes (2.5–4.5 MΩ) were filled with internal saline containing the following: 9 mM KCl, 10 mM KOH, 120 mM K-gluconate, 3.48 mM MgCl_2_, 10 mM HEPES, 4 mM NaCl, 4 mM Na_2_ATP, 0.4 mM Na_3_GTP, and 17.5 mM sucrose, pH adjusted to 7.25. TBB (CK2 inhibitor) and oxotremorine-m (muscarinic agonist) were purchased from Tocris. H89 (PKA inhibitor) was purchased from Abcam. Patch-clamp recordings were performed in current-clamp mode (capacitance cancellation switched off) using an EPC-10 amplifier (HEKA Electronics). Membrane voltage and current were filtered at 3 kHz, digitized at 10 kHz, and acquired using Pulse software (HEKA Electronics). After whole-cell patching in voltage clamp, series resistance was measured and bridge compensated once in current clamp. Before recording in current clamp, a bias current was applied to prevent spontaneous spike activity and to set baseline voltage to −70 mV. Intrinsic plasticity was monitored during test periods by injection of brief (500 ms) depolarizing current pulses adjusted to evoke four to eight spikes. The amplitude of the current needed to achieve this spike output did not differ between WT and SK2 knock-out mice (*p* > 0.05) and also did not differ in the presence of H89 or TBB in the bath (*p* > 0.05). The spike count was taken as a measure of excitability. Input resistance (*R_i_*) was measured by injection of hyperpolarizing test currents (100 pA, 100 ms) and was calculated from the voltage transient toward the end of current injection.

### Genetically modified mice

SK2KO mice were originally described in Bond et al. (2004). They were generated on the C57Bl/6 background (mice were obtained from John P. Adelman, OHSU). T305D mice were originally described in Elgersma et al. (2002; mice were obtained from Y. Elgersma, Erasmus MC, Rotterdam, The Netherlands) and were likewise generated on the C57Bl/6 background. All mice that were not genetically modified were C57Bl/6 mice.

### Data analysis

Data obtained from the pyramidal cell recordings *ex vivo* were analyzed using Pulsefit (HEKA Electronics), Igor Pro software (WaveMetrics), and R. For statistical analysis, we used the paired Student’s *t* test and the Mann–Whitney *U* test, when appropriate. Baseline periods were an average of 5 min prior to stimulations, and post periods were calculated in each group as an average of the relevant measurements 23–27 min following stimulation. Within group measures compared the difference between an individual cell’s baseline and post, using a paired Student’s *t* test. Between group measurements of more than two groups used the Kruskal–Wallis test, and Mann–Whitney *U* tests were used to directly compare groups. In all figures, the values shown represent the mean ± SEM.

## Results

**Figure 1.**
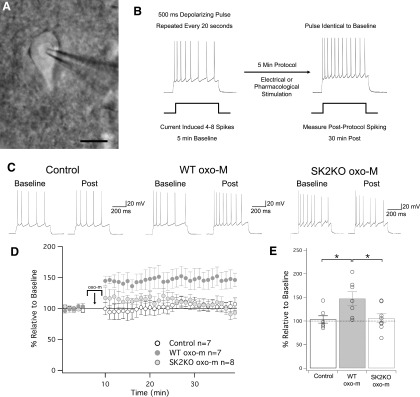
Muscarinic signaling induces changes in intrinsic excitability through SK2 channels. ***A***, Differential interference contrast (DIC) image of patch-clamp recording from a layer 2/3 pyramidal neuron in a slice prepared from S1 cortex; scale bar = 10 μm. ***B***, Test protocol for intrinsic plasticity. A stable period of 5 min of baseline was collected, where 500-ms pulses were delivered at 0.05 Hz, eliciting four to eight spikes. Following this is a 5-min induction protocol, where oxo-m (or electrical stimulation in later figures) is applied to the slice. Following induction, cells are monitored with the same test pulses used in baseline. ***C***, Example traces of baseline and postinduction for ACSF wash-on (control), oxo-m wash-on, and oxo-m wash-on in SK2KO cells. ***D***, Time graph for changes in spiking relative to baseline for all three groups. oxo-m/ACSF wash on occurs from minute 5 to 10. ***E***, Bar graph of each groups change in spiking relative to baseline. oxo-m significantly increased from baseline (*p* = 0.017), while ACSF and oxo-m in SK2KO mice did not (*p* = 0.68 and *p* = 0.57, respectively). Additionally, the increase observed in oxo-m was significantly greater than the changes observed in the ACSF and SK2KO oxo-m groups (*p* = 0.021 and *p* = 0.042, respectively). *p < 0.05.

To monitor changes in the membrane excitability of L2/3 pyramidal neurons, we performed whole-cell patch-clamp recordings in slices (350 μm thick) from S1 cortex of P25–P40 mice at near-physiological temperature (31–34°C). Excitability was measured in current-clamp mode by injecting brief depolarizing currents (500 ms) that were adjusted to evoke four to eight spikes during the baseline. In the test periods before and after any experimental manipulation, these current steps were delivered at 0.05 Hz. The number of spikes evoked by these constant depolarizing currents was taken as a measure of excitability. Under control conditions, in the absence of drug application or electrical stimulation, the spike count remained stable (103 ± 8% of baseline ± SEM, *n* = 7, *p* = 0.68; Fig. 1).

Bath application of the muscarinic agonist oxotremorine-m (oxo-m; 7 μM) for 5 min caused a lasting increase in the spike count (147 ± 14% of baseline ± SEM, *n* = 7, *p* = 0.017; Fig. 1). With the recording chamber design, slice thickness and perfusion speed used, oxo-m (7 μM) wash-out is complete after approximately 25 min (Rinaldo and Hansel, 2013). Thus, the lasting increase in excitability is likely due to intrinsic plasticity, and does not depend on the continuous presence of oxo-m in the bath. All three SK subunits (SK1, SK2, and SK3) are expressed in cortical pyramidal neurons (determined in rat; Gymnopoulos et al., 2014). In light of previous findings of a specific involvement of SK2 channels in intrinsic plasticity (Lin et al., 2008; Belmeguenai et al., 2010), we tested whether oxo-m bath application upregulates excitability in mice deficient of SK2 channels (SK2 null mice; Bond et al., 2004). Indeed, oxo-m (7 μM) did not enhance the spike count in L2/3 pyramidal neurons from SK2 null mice (106 ± 9% of baseline ± SEM, *n* = 8, *p* = 0.57; Fig. 1).

**Figure 2.**
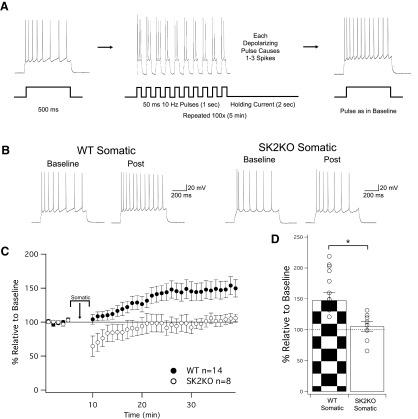
Induction of intrinsic plasticity through somatic depolarization is SK2 dependent. ***A***, Protocol for inducing changes in intrinsic plasticity, modeled off of Mahon and Charpier (2012). As before, a test pulse of 500 ms is repeated at 0.05 Hz in order to induce four to eight spikes. Following a 5-min baseline, the induction protocol injects depolarizing current at 10 Hz (50-ms pulses), evoking one to three spikes per pulse for 1 s, followed by 2 s of holding current. This 3-s sequence is repeated 100 times for a total of 5 min, and then test pulses identical to baseline are again delivered to monitor for changes in excitability. ***B***, Example traces of WT neurons and SK2KO neurons before and after the induction protocol. ***C***, Time graph for changes in spiking relative to baseline, induction protocol occurs from minute 5 to 10. ***D***, Bar graph for change in spiking relative to baseline. WT significantly increased from baseline, while SK2KO did not (*p* = 0.0028 and *p* = 0.55, respectively). The increase observed in WT cells was significantly greater than the changes observed in the SK2KO group (*p* = 0.044). *p < 0.05.

Intrinsic plasticity is triggered by repeated injection of depolarizing current pulses in cortical pyramidal neurons of intact rodents (Paz et al., 2009; Mahon and Charpier, 2012). To test whether SK2 channel plasticity can be elicited in a similar activity-dependent manner in L2/3 pyramidal neurons in slice, we applied a stimulus protocol, in which 10 depolarizing current pulses (50-ms duration) were delivered at 10 Hz, followed by 2 s of holding current. The depolarization amplitude was adjusted to evoke one to three spikes during each of these pulses, and was repeated 100 times for a total of 5 min of stimulation. Application of this depolarization protocol resulted in an increase in the spike count in pyramidal neurons from wild-type littermate controls (148 ± 13% of baseline ± SEM, *n* = 14, *p* = 0.003; Fig. 2) that was not seen in pyramidal neurons from SK2 null mice (105 ± 8% of baseline ± SEM, *n* = 8, *p* = 0.55; Fig. 2).

**Figure 3.**
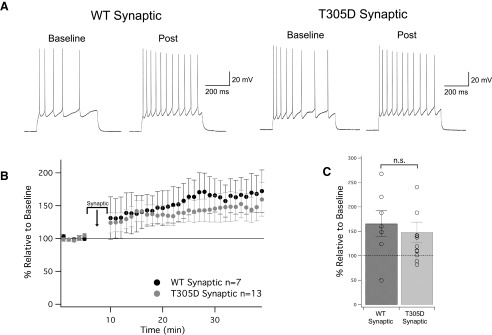
Induction of intrinsic plasticity is CaMKII independent. ***A***, Example traces of WT neurons and T305D neurons before and after the induction protocol. The induction protocol was modified from somatic depolarization where an extracellular stimulus was used in lieu of somatic stimulation (further details in text). ***B***, Time graph for changes in spiking relative to baseline, induction protocol occurs from minute 5 to 10. ***C***, Bar graph for change in spiking relative to baseline. WT and T305D both significantly increased from baseline (*p* = 0.048 and *p* = 0.040, respectively). The comparative increase between these two groups was not significantly different (*p* = 0.27). n.s. = nonsignificant.

We next tested for an involvement of calcium-calmodulin-dependent kinase II (αCaMKII) in SK2 channel plasticity, because CaMKII is one of the most abundant activity and calcium sensors in neurons. To this end, we used CaMKII T305D mice (Elgersma et al., 2002), in which Thr305 is substituted by a negatively charged aspartate. This genetic manipulation prevents calcium/calmodulin binding and thus keeps αCaMKII inactive (Rich and Schulman, 1998). Since αCaMKII is located in dendritic spines and the postsynaptic density (PSD; Kennedy, 2000), this kinase is best activated by synaptic stimulation. We therefore altered the stimulation protocol by replacing injection of depolarizing current pulses with trains of synaptic stimulation (10-Hz stimulating pulses for 1 s, followed by 2 s of no stimulation, repeated 100 times). An extracellular electrode was placed in layer 1 near the apical dendrite of the recorded cell, and stimulation strength was adjusted in order to evoke a postsynaptic spike >50% of the time. Under these activation conditions, intrinsic plasticity is efficiently triggered in wild-type controls (165 ± 27% of baseline ± SEM, *n* = 7, *p* = 0.048; Fig. 3). Synaptic stimulation also triggered intrinsic plasticity in T305D mice (148 ± 21% of baseline ± SEM, *n* = 13, *p* = 0.040; Fig. 3). Thus, αCaMKII activation is not needed for this type of plasticity.

**Figure 4.**
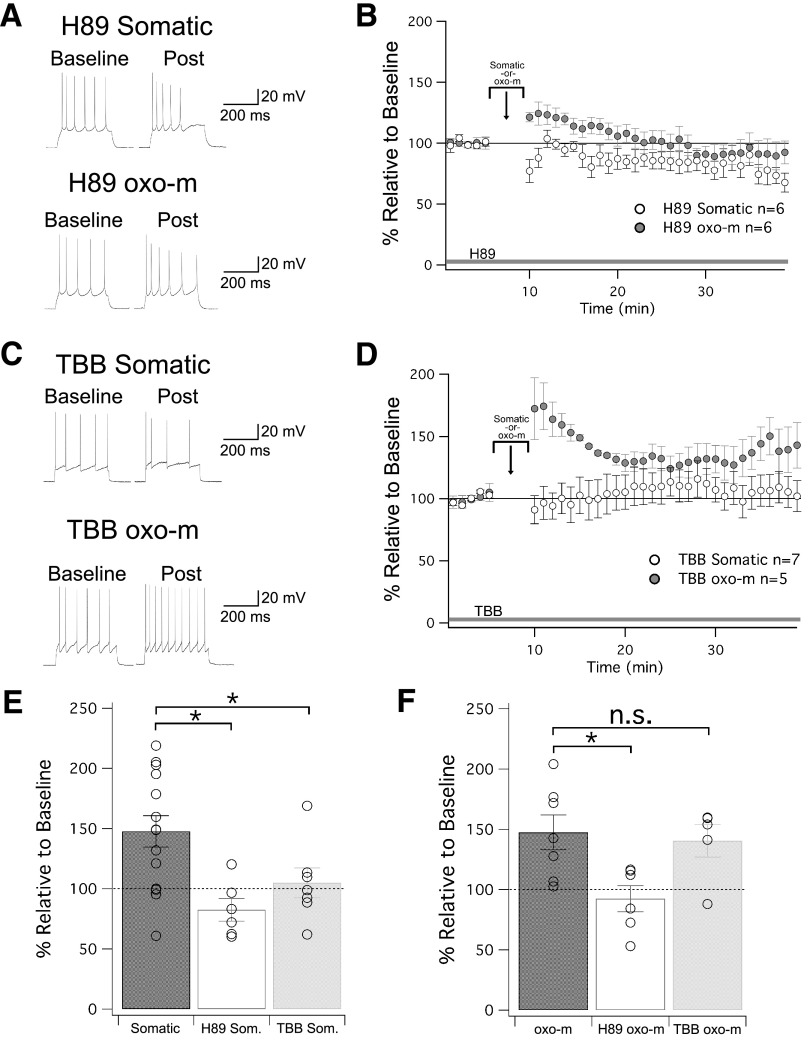
The role of PKA and CK2 in regulating SK2 channels in intrinsic plasticity. ***A***, Example traces for cells treated with H89, a PKA inhibitor that was present in the bath for the duration of the experiment. ***B***, Time graph for changes in spiking relative to baseline. Either somatic depolarization or oxo-m was applied from minute 5 to 10. ***C***, Example traces for cells treated with TBB, a CK2 inhibitor that was present in the bath for the duration of the experiment. ***D***, Time graph for changes in spiking relative to baseline. Either somatic depolarization or oxo-m was applied from minute 5 to 10. ***E***, Bar graph for depolarization-induced changes in spiking relative to baseline. Both H89 and TBB bath application prevented intrinsic plasticity. ***F***, Bar graph for oxo-m-induced changes in spiking relative to baseline. H89, but not TBB, prevented intrinsic plasticity; *p < 0.05. n.s. = nonsignificant.

Identifying SK2 as a mediator of intrinsic plasticity, we sought to determine if changes in plasticity may result from an internalization of SK2 channels that, in CA1 hippocampus, has been shown to depend on the activation of PKA (Lin et al., 2008). Bath application of the PKA inhibitor H89 (10 μM) indeed prevented an upregulation of membrane excitability upon repeated injection of depolarizing current pulses (82 ± 9% of baseline ± SEM, *n* = 6, *p* = 0.12; Fig. 4). An alternative pathway for SK channel plasticity, initially demonstrated in non-neuronal cell lines expressing SK2 channels, is the activation of CK2, which reduces the calcium sensitivity of SK2 channels (Allen et al., 2007). In CA1 hippocampal pyramidal neurons, M1 mAChRs may inhibit SK2 channels via CK2 activation (Giessel and Sabatini, 2010; but see Buchanan et al., 2010). In addition, in cerebellar Purkinje cells, depolarization-triggered SK2 plasticity is CK2 dependent (Belmeguenai et al., 2010). In the presence of the CK2 inhibitor TBB (2 μM) in the bath, repeated injection of depolarizing current pulses did not cause an increase in excitability (105 ± 12% of baseline ± SEM, *n* = 7, *p* = 0.71; Fig. 4). These findings show that SK2-mediated intrinsic plasticity depends on the activation of PKA and CK2, and thus involves both a modification of the calcium sensitivity of SK channels as well as SK2 channel trafficking. As an additional measure, we tested the effect of muscarinic activation during PKA inhibition. While PKA inhibitor H89 (10 μM) was in the bath, transient application of oxo-m was unable to induce a change in excitability (92 ± 11% of baseline ± SEM, *n* = 6, *p* = 0.51; Fig. 4). However, when TBB (2 μM) was in the bath, oxo-m was still able to enhance excitability (140 ± 14% of baseline ± SEM, *n* = 5, *p* = 0.04; Fig. 4). The amplitude of the excitability increase was not different from that observed when oxo-m alone was bath applied without TBB (*p* = 0.749). This illustrates that somatic stimulation capable of inducing changes in intrinsic plasticity converges with muscarinic activation upstream of SK2 channels onto signaling pathways that include the activation of PKA, but not those that include the activation of CK2. Neither H89 nor TBB alone caused changes in membrane excitability. This was measured by comparing the amplitude of depolarizing currents needed to adjust spike activity (four to eight spikes) during the baseline (control: 452.4 ± 46.3 pA, *n* = 36/H89: 370.0 ± 96.6 pA, *n* = 12/TBB: 480.0 ± 114.0 pA, *n* = 12/for comparison, SK2-KO: 530.0 ± 64.6 pA, *n* = 16/all group comparisons: *p* > 0.05).

**Figure 5.**
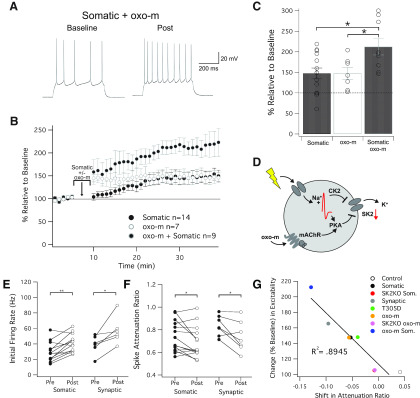
Intersection of somatic and muscarinic activation. ***A***, Example trace of a cell that received somatic depolarization while oxo-m was in the bath. ***B***, Time graph for changes in spiking relative to baseline, somatic, and oxo-m stimulation occurs at minute 5 to 10. ***C***, Bar graph for change in spiking relative to baseline. Combined stimulation was significantly different from baseline (*p* = 4.9 × 10^−4^), and significantly different from somatic or oxo-m stimulation alone (*p* = 0.035 and *p* = 0.044, respectively). ***D***, Diagram for how somatic depolarization and muscarinic pathways overlap and interact with SK2 channels. ***E***, Difference in initial firing rate for somatic and synaptic induction protocols. Both groups increased their firing rate per sweep from baseline to post (*p* = 0.002 and *p* = 0.020, respectively). ***F***, Spike attenuation ratios for somatic and synaptic induction protocols. The spike attenuation ratio is a ratio of the spiking that takes place in the first half of the sweep, and decreases for both somatic and synaptic cell groups (*p* = 0.045 and *p* = 0.034, respectively). ***G***, Shift in attenuation ratio is strongly correlated to change in intrinsic excitability. All cells from groups which had ACSF in the bath during baseline and post are plotted, indicating a strong connection between firing later in sweeps and intrinsic plasticity (*p* = 3.8 × 10^−4^).

Finally, we asked whether the amplitude of intrinsic plasticity changes when activity dependent and muscarinic application are co-applied. We therefore applied our somatic depolarization protocol (10 Hz, 1 s on/2 s off, for 5 min), while oxo-m (7 μM) was applied in the bath. This protocol caused a significant increase in spike count (212 ± 20% of baseline ± SEM, *n* = 9, *p* = 4.9 × 10^−4^; Fig. 5) that was larger than either manipulation alone (148 ± 13% and 147 ± 14% for somatic and oxo-m, *p* = 0.035 and 0.044, respectively), but did not exceed the arithmetic sum of excitability changes observed after these manipulations (mathematically 195 ± 13%, comparison to actual combination observed yields; *p* = 0.49). This result suggests that oxo-m application (at a near-maximally effective concentration; see Dennis et al., 2016) and repeated depolarization alone, respectively, do not achieve saturation of SK2 channel plasticity, respectively, and that there are no synergistic activation effects either. However, muscarinic activation may help to enhance intrinsic plasticity under conditions of elevated activity and may in this way contribute to effect maximization. Somatic depolarization also caused an increase in *R_i_* (126.2 ± 5.0% of baseline, *n* = 14, *p* < 0.001) that was not observed with statistical significance with oxo-m application (113.8 ± 12.0%, *n* = 7, *p* = 0.21). Somatic depolarization in the presence of oxo-m caused a change in *R_i_* that was in the range of that seen with somatic depolarization alone (122.1 ± 3.4%, *n* = 9, *p* = 0.004).

To further classify the effect of somatic depolarization and muscarinic activation, we examined the effects of these conditions on metrics within individual responses to injection of depolarizing current pulses. We first measured the instantaneous firing rate of the first four spikes within responses, both before and after somatic/synaptic tetanization. The firing rate was calculated for spikes 1–4 of an individual sweep, and averaged over the baseline and post periods of each recording. The somatic depolarization group saw a significant increase in the average initial firing rate following stimulation (29.7 ± 3.5 Hz increasing to 40.8 ± 3.2 Hz, rate ± SEM, *n* = 13, *p* = 0.002; Fig. 5*E*). Similarly, the synaptic depolarization group saw a significant increase in the average initial firing rate following stimulation (39.0 ± 5.0 Hz increasing to 57.2 ± 7.2 Hz, rate ± SEM, *n* = 6, *p* = 0.02; Fig. 5*E*).

We then sought to measure spike frequency adaptation within individual sweeps. Frequency adaptation causes an attenuation of spike firing toward the end of the depolarizing pulse. This parameter was measured by calculating the number of spikes within the first 250 ms of depolarization and dividing it by the total number of spikes in each sweep. Therefore, a ratio of 1 would indicate all spiking takes place in the early phase (first 250 ms) of a pulse, and a ratio of 0.5 would indicate spiking is uniform throughout the 500 ms of depolarization. As before, baseline and post values were averaged from every sweep within the cell. The somatic depolarization group saw a significant downward shift in spike attenuation (−0.053 ± 0.024, shift in attenuation ± SEM, *n* = 14, *p* = 0.045), indicating an increase in later phase firing. The synaptic stimulation group likewise saw a significant downward shift in attenuation (−0.096 ± 0.035, shift in attenuation ± SEM, *n* = 7, *p* = 0.034). Lastly, we looked at each group of cells which did not have a drug in the bath for the whole experiment. When we plotted their shift in spike attenuation against their increase in intrinsic excitability, we found a strong correlation between these two factors (*R*^2^ = 0.8945, *p* = 3.8 × 10^−4^). Given the connection between SK channels and late phase firing, this result offers additional support that our observed changes in excitability are strongly dependent on changes in SK2 channel function and/or expression levels. Unexpectedly, SK2 null mice showed a higher attenuation ratio during the baseline (0.82 ± 0.03, *n* = 16) than wild-type littermate controls (0.72 ± 0.02, *n* = 36, *p* = 0.039). This effect could result from an increase in spike frequency during the early phase of the depolarization phase as a consequence of the complete absence of SK2 channels in these mice. Alternatively, it is conceivable that compensatory mechanisms with different adaptation kinetics surfaced in SK2 null mice. Neither H89 (0.77 ± 0.04, *n* = 12, *p* = 0.374) not TBB (0.76 ± 0.05, *n* = 12, *p* = 0.501) changed the baseline adaptation ratio as compared with wild-type controls.

## Discussion

The main finding of this study is that activation of mAChRs in L2/3 cortical pyramidal neurons triggers intrinsic plasticity, and that it does so in an SK2 channel-dependent way. Because of their role in the mAHP, SK channels are particularly efficient in regulating membrane excitability over several hundred milliseconds (Sah, 1996; Stocker et al., 1999; Pedarzani et al., 2001; Edgerton and Reinhart, 2003). This feature puts these channels into a position to control plateau potentials (Cai et al., 2004). In addition, their functional downregulation promotes the occurrence of spike bursts, rather than just individual spikes (Ohtsuki and Hansel, 2018).

Our findings on the cellular mechanisms involved in SK channel plasticity align well with previous observations made in studies using transfected non-neuronal cell lines and pyramidal neurons in CA1 hippocampus. SK2 plasticity may arise from PKA-dependent channel internalization (Lin et al., 2008), or from CK2-dependent changes in calcium sensitivity (Allen et al., 2007). As intrinsic plasticity was prevented upon bath application of the PKA inhibitor H89 as well as the CK2 inhibitor TBB, it seems that both regulatory pathways are involved in SK plasticity in L2/3 cortical pyramidal neurons as well. While no detailed information on the cellular localization of the players involved in SK2 plasticity is available, including mAChRs, PKA, CK2, and SK2 channels themselves, our observations suggest that they are located sufficiently close to each other to engage in such modulatory interaction. As in these recordings, membrane excitability was assessed during the test periods before and after tetanization by the somatic injection of depolarizing current pulses, we can conclude that the plasticity location in these experiments is in or near the soma. (Note, however, that SK2 plasticity may also selectively occur in neuronal dendrites; see Ohtsuki et al., 2012).

The role of CK2 in the acute modulation of SK channels by M1 mAChRs has been subject of controversy. While Giessel and Sabatini (2010) reported that M1 receptors inhibit SK channels via CK2 activation, Buchanan et al. (2010) showed that CK2 receptors are not involved, but that instead PKC activation downregulates SK channels (see also Maylie and Adelman, 2010). Our findings show that CK2 is mechanistically able to downregulate SK2 channels, but that, at least under our conditions, this is not a consequence of mAChR activation. It remains possible, however, that such a regulatory action is more efficiently driven by the specific activation of M1 receptors. Finally, we observed that inhibition of αCaMKII does not affect intrinsic plasticity, similar to previous findings in Purkinje cells (Belmeguenai et al., 2010). Our experiments do not rule out a possible role for PKC in SK channel plasticity as suggested by Buchanan et al. (2010).

Intrinsic plasticity does affect behavioral learning. While no data are available for types of learning that specifically involve the neocortex, hippocampus-dependent and/or amygdala-dependent forms of learning, such as spatial learning in the Morris water maze, contextual and cued fear-conditioning as well as trace eyeblink conditioning are impaired upon SK2 overexpression (Hammond et al., 2006; McKay et al., 2012). Thus, SK2 regulation to an optimal level constitutes a critical component in learning tasks. Intrinsic plasticity may serve functions in learning that go beyond a role that is merely complementary to its synaptic counterpart (Marder et al., 1996; Hansel et al., 2001; Triesch, 2007; Debanne, 2009). In a recent perspective paper (Titley et al., 2017), we have argued that purely synaptic theories of learning are insufficient to explain the integration of neurons into functional ensembles that in the context of learning become “mnemic traces” (Semon, 1904), or memory engrams. In this interpretation, intrinsic plasticity may provide a mechanism to enhance the probability of neuronal spike firing upon activation, via lowering the spike threshold and/or altering the frequency versus input (F-I) function. SK channel plasticity efficiently serves this purpose. In addition, it enhances temporal precision of spike firing in response to synaptic input (Sourdet et al., 2003) and adjusts pauses in spike firing (Grasselli et al., 2016). Muscarinic inhibition of SK channels, both transiently and long lasting, may thus drive the participation of individual neurons in memory engrams. It is conceivable that this consequence of cholinergic signaling provides the cellular mechanism to link arousal and attention to the emergence of functional neuronal ensembles. Enhanced spike firing rate is expected to increase the signal-to-noise ratio of neuronal activity, because the standard deviation of spike counts typically increases only as the square root of the mean spike count (Heggelund and Albus, 1978; Softky and Koch, 1993). An ensemble-promoting effect is also suggested by reports that show that in hippocampus and visual cortex cholinergic modulation enhances the power of γ frequency oscillations and response synchronization among interconnected neurons (Rodriguez et al., 2004; Betterton et al., 2017). Notably, attention and cholinergic signaling can also inhibit pyramidal neurons (Gulledge and Stuart, 2005) and reduce interneuronal correlations (Cohen and Maunsell, 2009; Eggermann et al., 2014; Runfeldt et al., 2014). This range of even opposing effects might well be related to differences in types of neurons studied, local concentration of acetylcholine reached, distinct ensembles affected (e.g., distinct center-surround effects) and/or activation of inhibitory networks (Müller and Singer, 1989). Cellular physiological studies as well as electrical and optical recordings from intact animals will be required to assess the conditions under which these different consequences of cholinergic signaling take place.

## Synthesis

Reviewing Editor: Siegrid Löwel, University of Goettingen Decisions are customarily a result of the Reviewing Editor and the peer reviewers coming together and discussing their recommendations until a consensus is reached. When revisions are invited, a fact-based synthesis statement explaining their decision and outlining what is needed to prepare a revision will be listed below. Note: If this manuscript was transferred from JNeurosci and a decision was made to accept the manuscript without peer review, a brief statement to this effect will instead be what is listed below.

In the manuscript “Muscarinic downregulation of SK2-type K+ conductances promotes intrinsic plasticity in L2/3 pyramidal neurons of the mouse primary somatosensory cortex”, the authors describe that mAChR activation triggers an increase in membrane excitability in mouse S1 layer 2/3 pyramidal neurons. They found that the number of action potentials triggered by somatic current injections increased, and that repeated somatic depolarizations or synaptic activation causes a similar increase in membrane excitability. The authors suggested a mechanistic link of the increases in membrane excitability by showing that mAChR activation and repeated somatic depolarizations fail to increase membrane excitability in SK2 KO mice. Furthermore, pharmacological inhibition of PKA or CK prevented the increase with mAChR activation and somatic depolarizations, while a CaMKIIalpha knock-in mouse does not affect the synaptically triggered increase in excitability.

The findings are interesting, but both reviewers feel that they are not yet sufficiently linked. The abstract and title is misleading and unclear in its statements: e.g. no experiment directly shows a reduction in SK2 conductances as stated in the title. Which SK2 isoforms do the authors refer to in the abstract? The authors mention the modulation of SK2 by these two kinases, but do not provide experimental evidence that such modification actually occurs. Is SK2 localized where this plasticity is expressed? Is SK2 linked to mAChRs or at least colocalized with? The AP waveform in Fig. 1 for SK2 KO appear different than for control mice. H89 and TBB cause a decrease in excitability with repeated somatic depolarizations and changes in the AP waveform (TBB). These observations uncover some additional effects of the drugs and SK2 KO, which are expected as likely multiple signaling pathways are affected. However, they raise the concern that the observations may be secondary rather than a primary effect on SK2 function.

We therefore suggest that you take the above mentioned concerns into account, check off-target effects of H89 and TBB by evaluating the effects of the drugs alone, report shape parameters of the action potentials, moderate your conclusions about underlying mechanisms, and rephrase the title accordingly (we suggest to replace the word “conductance” by the word “channel”).

## Author Response

Point-by-point reply to the comments raised in the Synthesis Statement:

We would like to thank the reviewers and the editor for the constructive feedback, which helped us to improve the manuscript. All changes made in the revised paper are marked by italic, underlined text.

1)Sex of the species is not mentioned in the materials and methods section.

We prepared slices from mice of either sex. This is now stated in ‘Materials and Methods’ (p. 4). We noted the gender of all mice used (thus, this information is available), but we did not observe any gender-related differences.

2)The abstract and title is misleading and unclear in its statements: e.g. no experiment directly shows a reduction in SK2 conductances as stated in the title.

(Later in the synthesis): We ...suggest ....to replace the word “conductance” by the word “channel”.

In the revised manuscript title, we have replaced “downregulation” with “modulation” and ‘conductance’ with ‘channel’ (title page).

3)Which SK2 isoforms do the authors refer to in the abstract?

SK2 is one of three isoforms of the SK channel. This is explained later in the introduction, but for clarity, we now state in the abstract (p. 2): ‘....mice deficient of SK channels of the SK2 isoform’.

4)The authors mention the modulation of SK2 by these two kinases, but do not provide experimental evidence that such modulation actually occurs.

Studies on the biochemical pathways regulating SK2 channels that involve PKA and CK2 have been published several years ago by John Adelman and his group at the Vollum Institute. This work consisted of recordings from transfected non-neuronal cells (testing the basic mechanism) as well as CA1 hippocampal neurons. The labs of Bernardo Sabatini and Jack Mellor, respectively, expanded on this work, also in CA1 hippocampus. Due to the rich literature available, we did not study these basic regulatory mechanisms in our experiments, but instead focused on their involvement in plasticity of SK2 channels. In the revised version of the manuscript, we provide a better explanation including more specifics on the types of cells that were used in prior studies (p. 6+8).

5)Is SK2 localized where this plasticity is expressed? Is SK2 linked to mAChRs or at least co- localized with?

The co-localization has not been specifically tested, other than showing that all three SK channel isoforms as well as M1, M3 and M4 mAChRs are expressed in cortical layer 2/3 (Rossner et al., 1993; Gymnopoulos et al., 2014). Our measures of membrane excitability were based on somatic recordings, thus we know that this is one possible site of plasticity. The absence of plasticity effects from SK2 null mice clearly assigns these channels as mediators of the excitability change. We can thus conclude that the cellular machinery involved needs to be located at or near the soma. This is now better presented in the introduction (p. 3) and discussion (p. 8).

6)The AP waveform in Fig 1 for SK2 KO appear different than for control mice. H89 and TBB cause a decrease in excitability with repeated somatic depolarizations and changes in the AP waveform (TBB). These observations uncover some additional effects of the drugs and SK2 KO, which are expected as likely multiple signaling pathways are affected. However, they raise the concern that the observations may be secondary rather than a primary effect of SK2 function.

Actually, there is a certain amount of variability in the AP waveforms. It would be relatively easy to replace the traces shown with more coherent examples, but we do not want to make this step without editorial advice. We actually did now analyze spike attenuation ratios during the baseline recordings in wild-type, SK2 KO, H89 and TBB conditions. While both drugs did not have any effect on this AP waveform parameter, we found a significantly higher attenuation ratio in the baseline recorded from SK2 KO mice. This finding is now reported and discussed on p. 8 (top).

7)We...suggest that you....check off-target effects of H89 and TBB by evaluating effects of the drugs alone.....

In our recordings, we actually do have a parameter that reflects baseline effects on membrane excitability quite faithfully. In each recording, we injected depolarizing current to adjust (evoked) spiking to the desired amount. The lower this current amplitude, the higher the excitability (in all recordings, a negative bias current was applied to set Vm to -70mV). We did not find significant differences in the current amplitude selected to evoke spikes between the wild-type, SK2 KO, H89 and TBB groups (in none of all pairwise comparisons). This result is now documented on p. 6 (bottom).

8)We...suggest that you...moderate your conclusions about underlying mechanisms.... With the changes in the title and manuscript itself, this request is likely fulfilled.
